# The Curious Case of a Heterozygous Loss-of-Function PSEN1 variant associated with Early-Onset Alzheimer’s Disease

**DOI:** 10.21203/rs.3.rs-7222993/v1

**Published:** 2025-08-27

**Authors:** Inmaculada Sanjuan Ruiz, Lutgarde Serneels, Katleen Craessaerts, Alison Goate, Wim Annaert, Lucia Chavez Gutierrez, Yonggang Shi, Nasim Sheikh-Bahaei, Joanna C. Jen, Eliana Marisa Ramos, Mihaela Campan, Pamela M Ward, Shino Magaki, Kelly Bartlone, Harry V. Vinters, David W. Craig, John M. Ringman, Bart Strooper

**Affiliations:** Flanders Institute for Biotechnology (VIB); Flanders Institute for Biotechnology (VIB); Flanders Institute for Biotechnology (VIB); Icahn School of Medicine at Mount Sinai; Flanders Institute for Biotechnology (VIB); Flanders Institute for Biotechnology (VIB); University of Southern California; University of Southern California; University of California, Los Angeles; University of California, Los Angeles; University of Southern California; University of Southern California; UCLA; UCLA; UCLA; Beckman Research Institute of City of Hope; University of California, Los Angeles; Flanders Institute for Biotechnology (VIB)

**Keywords:** PSEN1, Loss-of-function, Alzheimer’s disease, γ-secretase

## Abstract

**Background:**

Over 300 mutations in *PSEN1* have been identified as causes of early-onset Alzheimer’s disease (EOAD). While these include missense mutations and a few insertions, deletions, or duplications, none result in open reading frame shifts, and all alter γ-secretase function to increase the long/short Aβ ratio.

**Methods:**

We identified a novel heterozygous *PSEN1* nonsense variant, c.325A > T, in a patient and his father, both presenting with EOAD, resulting in the substitution of lysine 109 with a premature stop codon at position (p.K109*). This produces a truncated 109 amino acid (aa) N-terminal PSEN1 fragment. Functional characterization was performed using overexpression models and a heterozygous mouse model (Psen1^K109*/+^).

**Results:**

In overexpression models, downstream ATGs serve as alternative starting codons, generating a > 37kDa and a > 27 kDa PSEN1 C-terminal fragment (PSEN1-CTF_A_ and PSEN1-CTF_B_, respectively) that retain the two catalytic aspartates of γ-secretase. Heterozygous Psen1^K109*/+^ mice exhibited subtle phenotypic defects, including reduced Pen2 expression and mild APP-CTF accumulation. Notably, aged mice demonstrated significantly increased Psen2 protein expression, potentially contributing to an elevated Aβ42/Aβ38 ratio.

**Conclusions:**

These findings indicate that *PSEN1* c.325A > T (p.K109*) is not a complete loss-of-function mutation. However, to what extent and by what mechanism it contributes to EOAD pathogenesis remains unclear.

## Background

In this case report, both a patient and his father exhibited dementia alongside amyloid plaque accumulation in the brain. Genome sequencing identified a novel *PSEN1* mutation.

Autosomal dominant Alzheimer’s Disease (ADAD) is a form of young onset dementia with a frequency estimated at 3.7/100.000(1). The disease is caused by dominantly inherited mutations in *PSEN1, PSEN2* or *APP* genes(2). Heterozygous carriers of *PSEN1* mutations present with the earliest age of dementia onset (mean of 43.6 ± 7.2 years).

While the cardinal features of classical AD are observed in patients with *PSEN1* mutations, they also suffer to various extents from additional atypical clinical phenotypes, such as myoclonus, seizures, pyramidal and extrapyramidal signs, and atypical neuropathology such as cotton wool amyloid plaques(3–6). The early onset, dominant inheritance of the disease, and additional neurological symptoms differ from sporadic AD, which is very common in the population. Although ADAD is rare, the impact of this disease is devastating, affecting individuals in the middle of their careers, and requiring many years of care by a partner, who is in many cases also responsible for the care of young children.

Presenilins (PSENs) are the catalytic core of the γ-secretase complex(7–11) which processes the amyloid precursor protein (APP) to generate Aβ peptides(7, 12). More than 300 mutations causing early onset AD (EOAD) (https://www.alzforum.org/mutations/psen-1) have been identified since the landmark publication from P. H. St George Hyslop(13) who identified the first mutations in what was then called the *S182* gene. The vast majority of these mutations are single nucleotide changes resulting in amino acid substitutions in the presenilin protein.

EOAD patients carrying *PSEN* mutations are almost always heterozygous, meaning that they also have a wild type presenilin allele that can compensate for most of its normal functions(14), including Notch signalling. A few insertions, deletions or duplications have been reported as well, but rarely cause open reading frame shifts (https://www.alzforum.org/mutations/psen-1). To date only two *PSEN1* mutations, E280A and A431E, have been documented to occur occasionally in homozygous carriers(15, 16). The debate whether mutations in PSEN1 cause ADAD by gain or loss of function is ongoing and has been reviewed recently(2).

Here we describe the first case of a subject and his father carrying a novel heterozygous nonsense *PSEN1* variant, c.325A > T (p.Lys109Ter), leading to the substitution of Lysine 109 with a premature stop codon (K109*). This potentially causes a significant truncation of the PSEN1 protein between transmembrane domains 1 and 2 (TM1 and TM2), which would result in the loss of ~ 75% of PSEN1 total open reading frame and the truncated mRNA transcript would be expected to undergo non-sense mediated decay.

Our interest was raised by this exceptional case which could be the first ever documented full loss of function *PSEN1* mutation associated with EOAD. We studied the functionality of the *PSEN1* c.325A > T variant (referred to as *PSEN1*^K109*^ from here on) for its effects on -secretase function *in vivo* and *in vitro*. Although we were able to demonstrate that this mutant generates two novel PSEN1 protein fragments in overexpression studies, the causal relationship between this novel mutation and ADAD remains uncertain.

## Methods

### Clinical assessment

The index patient was the only child of a father who had begun to have problems with short-term memory at age 39y, for instance, no longer being able to find his way when using a map. He later developed ataxia, dysarthria, and pseudobulbar affect and met criteria for dementia at age 46y. He died at age 56y. The father had one brother and one sister. His brother had no neurocognitive problems at age 68y. His sister had diabetes and blindness, probably caused by diabetic retinopathy by the age of 50y, and died of a myocardial infarction at 65y without known neurocognitive symptoms. The paternal grandmother of the index case is believed to have lived with dementia with symptom onset at 75y and death at 80y. The paternal grandfather died at age 70y from diabetes and multiple strokes but nonetheless was “pretty sharp until the end” as he continued to work from home until the time of his death.

### Human samples collection

DNA from the index case was initially screened in a CLIA-certified clinical laboratory using a NGS commercial panel of 37 genes known to be involved in neurodegeneration (Supplementary Table 1). The *PSEN1* variant reported by the clinical laboratory was confirmed independently in another academic laboratory using Sanger sequencing.

Formalin fixed tissue was available for the father which was sent to a CLIA certified molecular pathology lab. Primers were designed for Sanger sequencing to determine the presence or absence of the index case’s *PSEN1* c.325A > T heterozygous mutation. A peripheral blood sample from the index case was also submitted to the laboratory, which was used as a positive control.

Brain sections from the father were incubated with primary antibodies for amyloid pathology (1:150, Aβ42, Millipore AB5078P, rabbit polyclonal) and neurofibrillary pathology (1:200, AT8, Thermo Fisher, mouse monoclonal) followed by horse anti-mouse or horse anti-rabbit secondary antibody conjugated to horseradish peroxidase (MP7402 and MP7401; Vector Laboratories, Burlingame, CA). Antibody reactivity was visualized with N’N Diaminobenzidine as chromogen (no. SK-4100; Vector Laboratories) and counterstained with hematoxylin.

### Whole-genome sequencing (WGS)

The sample subsequently underwent whole genome sequencing at the American Genome Center at the Uniformed Services University. Whole-genome sequencing (WGS) was performed on extracted genomic DNA using a high-throughput sequencing platform following standard library preparation protocols. After demultiplexing and base calling, the resulting paired-end short reads were aligned to the GRCh38/hg38 human reference genome using a standard alignment tool (e.g., BWA-MEM). Post-alignment processing, which included marking of duplicate reads, base quality score recalibration, and read filtering, was conducted according to the Genome Analysis Toolkit (GATK) best practices pipeline. Variant discovery was carried out using GATK HaplotypeCaller (v4.1.1.0) in GVCF mode. Key parameters included --genotyping-mode DISCOVERY to capture both known and novel variants, --use-new-qual-calculator true to employ the updated quality score model, and --emit-ref-confidence GVCF to generate genome VCF (gVCF) files that record reference and variant confidence levels. Overclipped reads were filtered, and a minimum mapping quality of 20 was applied to ensure alignment accuracy. A minimum base quality threshold of 10 was used, and the base-quality-score-threshold parameter was set to 18 for likelihood calculations. Known variant sites from dbSNP (build 138 for GRCh38) were provided to improve the accuracy of variant calling and aid in differentiating true variants from sequencing artifacts. HaplotypeCaller parameters were further refined to balance sensitivity and specificity. The --standard-min-confidence-threshold-for-calling was set to 30 to ensure high-confidence variant calls, and the --pcr-indel-model NONE setting was applied to avoid unnecessary corrections for PCR-induced indels. Priors for expected heterozygosity were adjusted via --heterozygosity 0.001 and --indel-heterozygosity 1.25E-4, while the --max-alternate-alleles value was capped at 6 to limit complexity at multi-allelic sites. A wide range of genotype quality (GQ) bands was specified (--gvcf-gq-bands) to capture detailed confidence intervals for every site in the gVCF. The final output was saved in compressed gVCF format (.g.vcf.gz), with the VCF header defining filters (e.g., LowQual) and formats (e.g., AD, DP, GQ, GT, PL, SB) used to annotate each variant. Following variant calling, the resulting gVCFs were aggregated and jointly genotyped using GATK’s GenotypeGVCFs. Additional filtering steps—such as variant quality score recalibration, read depth thresholds, and strand-bias evaluation—were used to obtain a high-confidence set of variants. These variants were subsequently subjected to annotation, variant-impact assessment, and other downstream analyses.

### Sanger sequencing

Nested PCR was performed as follows: 50 ng of DNA was amplified with 0.5uM each 5’-GCGCCAAGCATGTGATCATG-3’ and 5’-GATGACATGCTGTAAAGAAAAGCCACAC-3’ primers in the first amplification PCR. Two microliters of the first PCR product were subsequently amplified with the 5uM each nested primers: 5’-TGTAAAACGACGGCCAGTGCTCTTTGTCCCTGTGACTCTCTG-3’ and 5’-CAGGAAACAGCTATGACAGAAAAGCCACACTGGCTTTGAG-3’ containing M13/Puc adapters. The first PCR amplification was done in a 50ul volume using IProof High-Fidelity Master Mix (Bio-Rad) at 98°C for 3 minutes, 20 cycles at 98°C (10sec), 61°C (10 sec), 72°C (15 sec), and 72°C (10 min). The nested PCR was done in 50ul volume using IProof High-Fidelity Master Mix (Bio-Rad) with a touch down protocol: 95°C (3 min), 20 cycles at 95°C (20 sec), decreasing annealing temperature from 70°C of a rate of −0.5°C/cycle (20 sec), 72°C (30sec), 25 cycles at 95°C (20 sec), 60°C (20 sec), 72°C (30 sec). The nested PCR product was purified using the Qiaquick PCR Purification kit (Qiagen) and eluted in 30ul of H2O following the manufacturer protocol. Five ul of the purified DNA (eluted in H2O) was sequenced using the Big Dye Terminator v3.1 Cycle sequencing Kit (ThermoFisher Scientidic) using the 0.15uM of the forward and reverse M13/Puc primers using the manufacturer suggested protocol. The sequenced products were clean up using DyeEx 2.0 Spin Kit (Qiagen) according with the manufacturer protocol. The whole purified sequenced DNA was mixed with 10ul of deionized formamide, denatured for 3 minutes at 95°C kept 5 minutes at 4°C and capillary electrophoresis was performed on the 3500 genetic analyzer (ThermoFisher Scientific). The sequencing files were visualized and interpreted with the Sequencher (Gene Codes).

### Animals

All mice were housed in cages enriched with wood wool and shavings as bedding, and given access to water and food ad libitum with a day/night schedule of 14/10h. C57Bl6 mice obtained and *Psen1*^+/−^ previously generated by our laboratory(17) were used in this study. All experiments were approved by the Ethical Committee for Animal Experimentation at the University of Leuven.

### Generation of *Psen1*^K109*^ Mice

Mice Psen1^em2Bdes^ mimicking the K109 stop mutation were generated using CRISPR-Cas9 technology by targeting exon 4 of the mouse *Psen1* gene (Fig. 2A). Ribonucleoproteins (RNPs) containing 0.3 μM purified Cas9HiFi protein (Integrated DNA Technologies, IDT), 0.6 μM cRNA tarteting 5’-AGCTTCTATACCCGGAAGGA − 3’, 0.6 μM trans activating crRNA (IDT) and 10ng/μl ssODN (5′-CCCCGTGACCCTCTGCATGGTCGTCGTCGTGGCCACCATCAAATCAGTCAGCTT CTAT**ACGCGT**TAGGATGGGCAGCTGTATGTATAAGTGCTTCGTTCTCTGGGCTGGTGTGGCTTTTCCTCGTAGCTTGTT3’) were injected into the pronucleus of C57Bl6J embryos by the Mouse Expertise Unit of KU Leuven. Positive founders were identified by PCR including MluI (bold indicated in the ssODN sequence) digestion and Sanger sequencing of the *Psen1* exon 4 region, confirmed correct targeting. The founder mouse was backcrossed once with C575Bl6J mice to avoid off target events in the colony and crossed with the App^em1Bdes^ mice in which Aβ sequence is humanized resulting in ~ 3-fold increase Aβ levels(18). Standard genotyping was performed by PCR with primers 5′- AGAGTCCGGCTTACTGAATG − 3′ and 5′- ATCATCACTGCCATCATCCC − 3′ followed by digestion of the PCR product with MluI, which resulted in 423 bp for the wildtype allele, and two fragments of 272 and 151 bp for the K109stop (K109*) knock in allele. The strain was kept on a homozygous *App*^*hu*/*hu*^ background and both females and males were included in the study. Time mating with heterozygous *Psen1*^K109*^ animals were set up for preparing neuronal cultures (E14.5) and photography (E17.5). The embryos were imaged with a Nikon SMZ25 microscope with an external camera.

### Cell Culture

#### Primary neuronal cultures

Primary neuronal cultures were prepared from wild-type *Psen1;App*^*hu*^ (WT); *Psen1*^K109*/+^;*App*^*hu*^ (K109*/+) and *Psen1*^K109*/K109*^;App^*hu*^ (K109*/K109*) mouse brains at embryonic day 14.5 and plated on poly-l-lysine (Sigma) coated 6 well plates, with 3 wells per embyo. Neurons were seeded in minimal essential medium (Invitrogen, catalog no. 31095–029) supplemented with horse serum, penicillin, and streptomycin (PenStrep, Invitrogen, catalog no. 15140–122). 4 h after plating, medium was replaced with Neurobasal medium (NB, Invitrogen, catalog no. 21103–049) supplemented with B27 (Invitrogen, catalog no. 17504–044), and PenStrep.

#### HeLa cell cultures

Hela Psen dKO cell line (kind gift of Prof. W. Annaert) was stably transduced using retroviral particles. For virus generation, HEK293T cells were transfected using TransIT-LT1 (Mirius Bio) according to the manufacturer’s protocol. The pMSCV transfer vector carrying the gene of interest was co-transfected with the helper plasmid pIk Ecopac (packaging vector encoding Gag and Pol) and pMD2.G (envelop vector encoding VSV-G). Medium containing the viral particles was collected after 48h and filtered (0.45 μm filter). For transduction, viral particles were diluted in polybrene containing medium (8 ng/μl, Sigma Aldrich). Cells were re-plated at density of 40–50% confluency 24h after the transduction and selection of transduced cells was achieved through antibiotic selection (puromycin, 5μg/ml, Sigma Aldrich). PSEN1 expression was validated by WB from 4 different stable pools of transduced cells.

#### Mouse Brain sampling

Mice were euthanized with an intraperitoneal injection of Dolethal (150–200 mg/kg) and brains were collected after transcardial perfusion with PBS. One brain hemisphere from wild-type *Psen1;App*^*hu*^ (WT); *Psen1*^K109*/+^*;App*^*hu*^ (K109*/+); C57Bl6 or *Psen1*^+/−^ mice was homogenized in Lysing Matrix D tubes (MP Biomedicals) containing 10 volumes of T-PER^™^ Tissue Protein Extraction Reagent (Thermo Fisher, catalog no. 78510) supplemented with cOmplete^™^ protease inhibitor cocktail (Roche) and PhosSTOP^™^ (Sigma) in a FastPrep-24^™^ homogenizer (MP Biomedicals) for 45 seconds at 6,5m/s. After samples were centrifuged 5 minutes at 5000g, supernatant was transferred to prechilled tubes (Beckman Coulter) and ultracentrifuged at 4°C for 1 hour at 100.000g in a Beckman Ultra Optima TLX ultracentrifuge. The resulting supernatant was used for western blot and Aβ ELISA measurements. The other brain hemisphere was post-fixed 24h in a formaldehyde solution 4%, buffered, pH 6.9 (Sigma), rinsed in PBS and embedded in 4% low melting point agarose (Thermo Scientific). Serial cuts of 40μm thickness were made with a vibratome (Leica) and stored in PBS with 0.01% sodium azide at 4°C until use for histology.

#### Mouse Histology

Brain sections were collected in free-floating conditions and washed twice in PBS. Antigen retrieval was performed by boiling three times the sections in 10 mM sodium citrate (pH 6) in a microwave. After letting sections cool down for 15min, they were rinsed three times in PBS and blocked for 1h at RT in a 5% donkey serum with 0,5% TX-100 PBS solution. Sections were incubated overnight at 4°C with primary antibodies against Aβ: 82E1 (IBL, 10323, 1/200) and Iba1 (WAKO, 019–19741, 1/500). The next day, sections were washed three times with 0.5% TritonX100-PBS and incubated with Alexa 488 Donkey anti-mouse IgG (Invitrogen, A21202, 1/500) and Alexa594 Donkey anti-rabbit IgG (Invitrogen, A21207, 1/500) for 1.5h at RT. After three washes with 0.5% TritonX100-PBS, sections were counterstained with DAPI (Sigma, D9542, 1/5.000) and after three final PBS washes, mounted on super frost microscope slides. Information about used antibodies can be found in Supplementary Table 4. Coverslips were mounted using Mowiol and sections were visualized on a Nikon AX confocal system.

#### RT-qPCR

The brain from 6.5m old mice was dissected. A small region from the right mouse hemisphere, containing middle cortex, hippocampus, thalamus and hypothalamus was microdissected. Total RNA extraction from this tissue piece was performed using the Qiagen RNeasy mini kit (Qiagen, #74104) according to the manufacturer’s instructions.

cDNA was synthesized from 200 ng total RNA using the Superscript II reverse transcriptase (ThermoFisher Scientific, #18064014).

cDNA was then used as template for Real-time semi-quantitative PCR reactions using the SensiFast Sybr No-Rox kit (Bioline, #BIO-98020) and containing either primers for the target genes or endogenous reference genes. All qPCR reactions were carried out on one single 384 well plate, 2 biological replicates per group. The number of technical replicates was 3 for all reactions.

Mean expression of three housekeeping genes was used for all normalizations (*Actb* and *Gapdh* and *HPRT*). The primer sequences of target gene and reference genes can be found in Supplementary Table 3. Cp (crossing points) were determined by using the second derivative method. Fold changes were calculated with the 2^−ΔΔCt^ method(19).

## ELISA

Aβ38, Aβ40 and Aβ42 levels from supernatant and brain homogenates were quantified on Meso Scale Discovery (MSD) 96-well plates using ELISA and antibodies provided by Dr. Marc Mercken (Janssen Pharmaceutica). Monoclonal antibodies JRFcAβ38/5, JRFcAβ40/28 and JRFcAβ42/26, which recognize the C terminus of Aβ species terminating at amino acid 38, 40 or 42, respectively, were used as capture antibodies. JRF/rAβ/2 (rodent specific antibody) or JRFAβN/25 (human specific antibody) labeled with sulfo-TAG were used as the detection antibodies. Human Aβ43 was measured using the commercially available amyloid-beta (1–43) high sensitivity ELISA kit from IBL (27710) according to the supplier’s protocol.

### Western blot

Total protein from neuronal lysates and brain homogenates was measured with a Biorad protein assay kit. 20 to 30μg of protein were mixed with sample buffer plus β-mercaptoethanol at a final concentration of 4% and denatured for 10 min at 70°C. Each sample was loaded on precast 10% or 4–12% NuPAGE Bis-Tris gel and electrophoresed at 150 V, 500 mA for 45min to 1h. Proteins were further transferred on 0.2-μm nitrocellulose membrane (Protran) at 25V for 1h. The membrane was blocked in Tris-buffered saline (TBS) with 0.1% Tween 20 and 5% nonfat milk. The primary antibodies were applied overnight at 4°C: Nicastrin (9C3, 1/4000)(20); mouse PSEN1-NTF (B19.3, 1/10.000)(21); human PSEN1-NTF (MKAD3.5, 1/1.000); mouse/human Psen1-CTF (Sigma Aldrich, MAB5232, 1/1.000); human PSEN1-CTF (Cell Signaling, D39D1, 1/1.000); mouse/human PSEN2-CTF (Cell Signaling, D30G3, 1/1.000); mouse/human PEN2 (B126.2, 1/1.000)(22); mouse/human APPC20 (B63.3; 1/5.000) (20); β-actin (Sigma, A5441, 1/20.000). Peroxidase-coupled secondary antibodies (Bio-Rad, 170–6515 and 170–6515, 1/10.000) were applied for 1h at room temperature. Information about used antibodies can be found in Supplementary Table 4. The blots were developed chemiluminescently using Western Lightening Plus (Perkin Elmer) and digitized by means of the ImageQuant LAS4000 mini reader. Quantification of bands was performed in Image Studio Lite version 5.2.

## Statistical Analysis

Statistical analysis was done using GraphPad Prism 10.2.3 software. Two-tailed unpaired Student’s t-test was performed to report statistical differences between two groups (Tables 1 and 2; Figs. 3B, 4B, 4D and S2A). One-way ANOVA followed by Tukey’s post-hoc test was performed to report statistical differences between 3 genotypes (Fig. 3D). Two-way ANOVA followed by Tukey’s post-hoc test was performed to report statistical differences between ≥ 2 genotypes in Aβ species production (Figs. 3A and 4C). Statistical significance was considered when *p* value < 0.05.

## Results

### Biomarker, imaging and genetic findings

The index patient is a male of European ancestry with a history of psoriatic arthritis since age 16y, who presented with subjective memory complaints at age 30y. At age 34y he had amnestic mild cognitive impairment, with a Clinical Dementia Rating (CDR)(23) score of 0.5. He continued to work until meeting criteria for dementia, CDR = 1, at age 36y at which time symptoms of delusions of persecution were apparent. His memory problems progressed and he confabulated and was increasingly anxious. Despite treatment with sertraline his psychiatric symptoms worsened by age 40y. The index patient’s father had developed short-term memory issues at 39y, followed by ataxia, dysarthria, pseudobulbar affect, and dementia at 46y. He died at 56y. The paternal grandmother likely had dementia from 75y until her death at 80y. The paternal grandfather died at age 70y from diabetes and multiple strokes but nonetheless was “pretty sharp until the end.”

**Figure 1:**
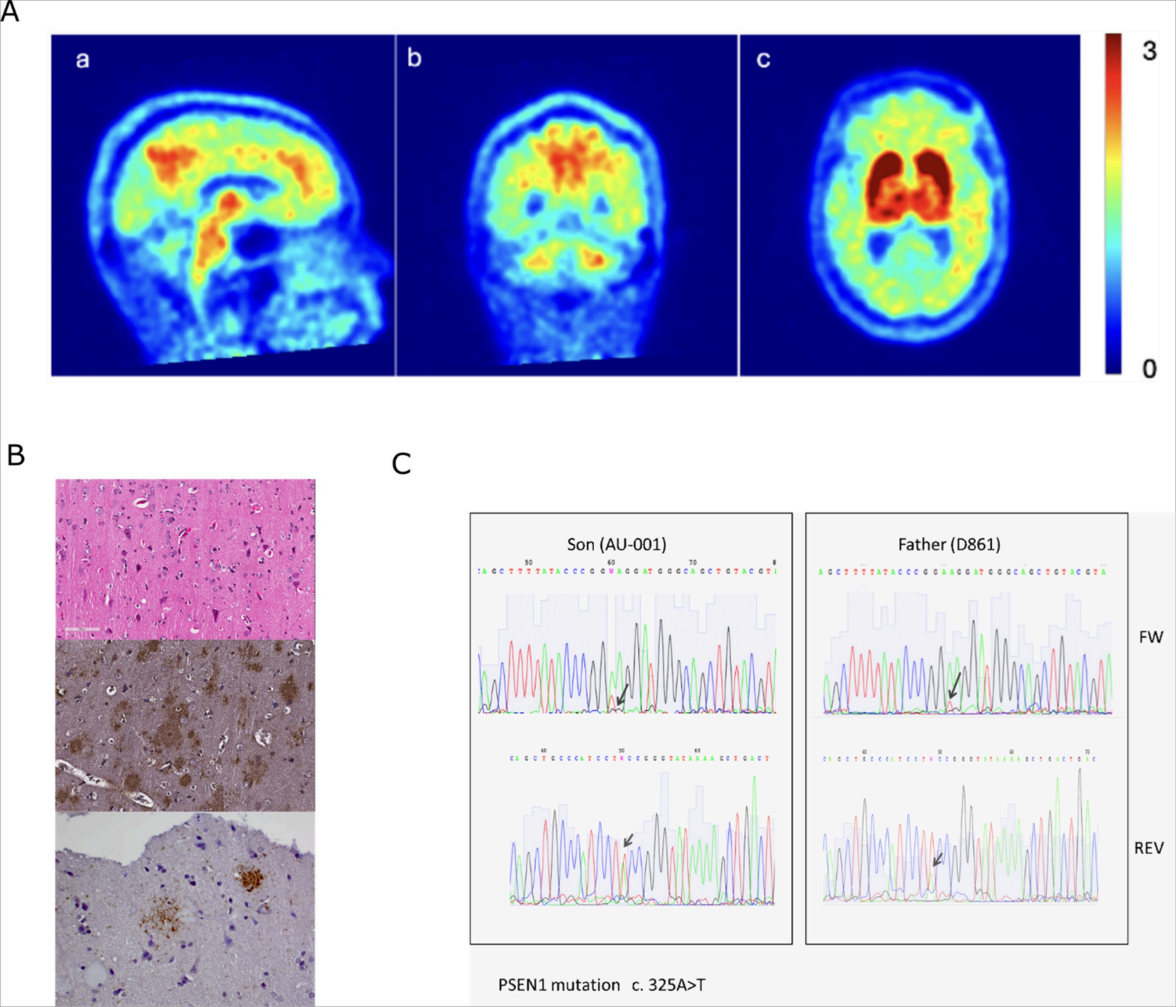
Pathological and genetic characterization of *PSEN1* c.325A>T (p.K109*) heterozygous carriers. **A)** ^11^C-PIB of the index patient at age 35 showed increased uptake and amyloid deposition in the posterior cingulate gyrus, precuneus, as well as in the brainstem and white matter of the cerebellum (a,b). Significant uptake was seen in the basal ganglia bilaterally including the caudate heads, lentiform nuclei and thalami (c). **B)** Histologic findings of the brain biopsy from the affected father of the index patient. Top – H&E stain at 20x showing cortical neurons, Middle – many amyloid deposits positive for Aβ42 (1/150, Millipore AB5078P) at 20x, Bottom - Neuritic plaques stained with AT8 (/1200, Thermo Fisher Scientific, MN1020) at 40x. **C)** Sanger sequencing to detect *PSEN1 c.325A*>*T* heterozygous mutation; forward primer (FW) sequencing index case and father and reverse primer (REV) sequencing index case and father. The arrows indicate the heterozygous substitution of a thymine for an adenine at position 325.

Biochemical and imaging biomarkers were compared between the index patient and matched controls in the Dominantly Inherited Alzheimer Network (DIAN) study. CSF Aβ42 and Aβ40 biomarker values were quantitatively, though not significantly lower than the means of age-matched non-mutation carriers/controls, while total tau and p-tau levels were significantly elevated (123 vs. 52, 115 vs 31.3 pg/ml, p ≤ 0.001) (Tables 1 and 2). Plasma Aβ42 levels were non-significantly higher (47 vs. 36 pg/ml), Aβ40 levels non-significantly lower (138 vs. 152 pg/ml), and Aβ42/Aβ40 ratio non-significantly higher (0.34 vs. 0.25) in the index case relative to 40 age-matched non-mutation carrying controls. Fluid biomarker values in the index case did not differ from those in CDR- and duration of symptom-matched ADAD mutation carriers in CSF (n = 27) and plasma (n = 31). Structural MRI showed mild atrophy with statistically larger volumes in the ventricles and of white matter hyperintensities, as well as smaller volumes in hippocampi, right precuneus, and thickness of the left caudal middle frontal gyrus relative to 39 non-mutation controls obtained from the DIAN cohort (p < 0.01). A PiB-positron emission tomography (PET) scan showed amyloid deposition consistent with AD with substantial signal in the basal ganglia, precuneus, brainstem, and cerebellum (Fig. 1A). The father underwent a right frontal cortical biopsy at age 48 that showed extracellular plaques staining positive for Aβ42 (Millipore AB5078P) and neurofibrillary pathology (Thermo Fischer, AT8), consistent with Alzheimer’s disease (Fig. 1B).

The index patient was found to be a heterozygous carrier of a c.325A > T *PSEN1* variant which is predicted to encode a premature stop codon at amino acid position 109 (p.Lys109Ter). No other potentially relevant genetic variants were found in 37 additional screened genes, known to be involved in neurodegeneration (see Supplementary Table 1). Whole genome sequencing revealed > 300 rare variants in the index patient (see Supplementary table 2). The *PSEN1* c.325A > T heterozygous mutation was detected in the father (Fig. 1C), but it was not present in his unaffected mother nor in a 68-year-old unaffected paternal uncle.

### Amino terminal protein fragment production from the PSEN1 c.325A > T mutation.

**Figure 2:**
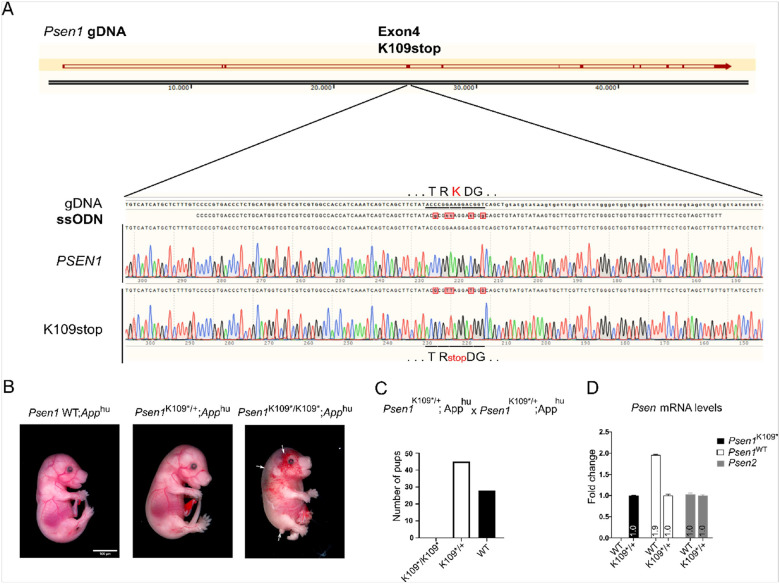
Generation and characterization of the *Psen1 c.325A*>*T* (*Psen1*^K109^*) mouse model. **A)** Upper panel represent the genomic structure of the mouse Psen1 gene. The location of the K109stop in exon 4 of the gene is indicated. Lower panel shows the ssDNA sequence used for the gene targeting including the 4 silent mutations introduced during gene editing and the sanger sequencing results of the founder mouse harboring one wild type allele and one allele coding for p.K109*. **B)** Images of *Psen1*^K109*^;App^*hu/hu*^ embryos at E17. Heterozygous mice are indiscernible from their wild-type littermates. Homozygous mice present a Notch-deficiency phenotype with defects in angiogenesis, neural tube formation and shortening of the tail anlage, indicated by white arrows. Scale bar: 100 μm. **C)** Bar plot showing the number of pups obtained at weaning age (3 weeks) from in total 14 litters from *Psen1*^K109*/+^*;App*^*hu/hu*^ matings. The observed distribution *Psen1*^K109*/K109*^;App^*hu/hu*^ (0%), *Psen1*^K109*/+^*;App*^*hu/hu*^ (62%), *Psen1*^*WT*^*;App*^*hu/hu*^ (38%) confirms the embryonic lethal phenotype in homozygous mice. **D)** Bar plot showing the mRNA levels of *Psen1*^*WT*^ (white bars); *Psen1*^K109*^ (black bars) and *Psen2* (grey bars) in wild-type or *Psen1* ^K109*/+^*;App*^*hu/hu*^ mice at 6.5 months of age (n=2 per genotype), assessed by RT-qPCR. Selective primers including the 5-nucleotide change introduced by the gene editing (panel A) were used to design selective PCR primer sets for the *Psen1*^*WT*^ and the *Psen1*^*K109*^ transcripts. Using the 2-ΔΔCt method, the data are presented as the fold change in gene expression normalized to the mean of three reference genes (Actb, Gapdh and Hprt) and relative to the expression in *Psen1*^K109*/+^*;App*^*hu/hu*^.

We generated a Psen1 c.325A > T knock-in mouse model (Fig. 2A). This strain was crossed with the humanized App knock-in mouse expressing human Aβ(18). Mice carrying the *Psen1* c.325A > T mutation are referred to as *Psen1*^K109*/+^ and *Psen1*^K109*/K109*^, respectively. At E17, heterozygous *Psen1*^K109*/+^;*App*^*hu/hu*^ mice were not discernibly different from their wild-type littermates. Strikingly, E17 homozygous *Psen1*^K109*/K109*^;App^*hu/hu*^ embryos (Fig. 2B) presented a Notch-deficiency phenotype characterized by defects in angiogenesis, neural tube formation and shortening of the tail anlage(7, 24–26). No viable *Psen1*^K109*/K109*^;App^*hu/hu*^ were born, indicative of the embryonic lethality of the phenotype (Fig. 2C). *Psen1* mRNA levels in 7-month-old viable *Psen1*^K109*/+^*;App*^*hu/hu*^ mice are half that of their wild-type littermates. *Psen2* mRNA levels are expressed at levels equal to those of their wild-type littermates (Fig. 2D).

**Figure 3:**
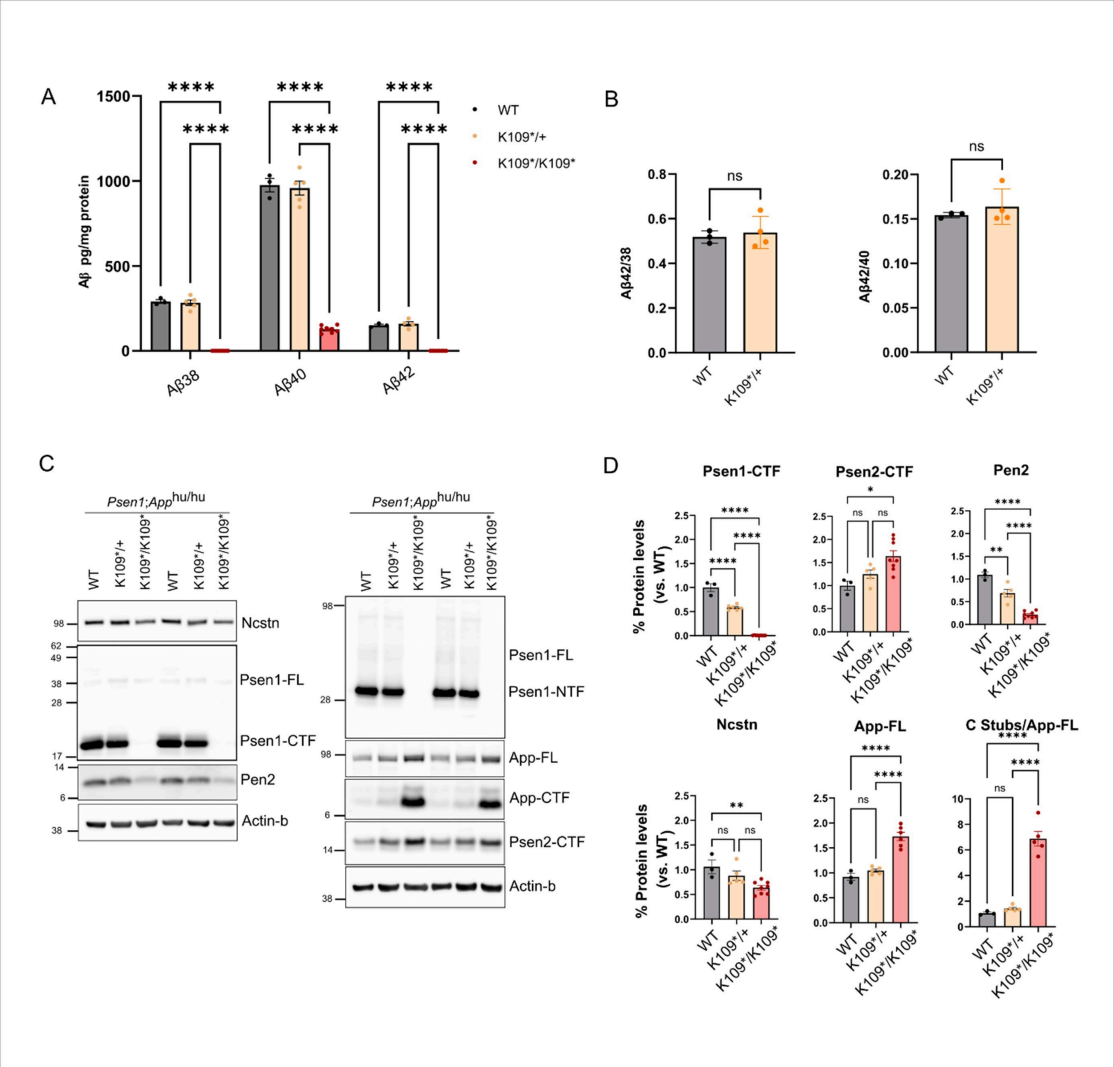
*Psen1* c.325A>T variant results in full loss of function in homozygous neurons. **A)** Bar plot showing the Aβ profile of wild-type (WT, black), heterozygous (K109*/+, orange) and homozygous (K109*/K109*, red) *Psen1*^K109*^;App^*hu/hu*^ neurons measured by MSD-ELISA from conditioned media. N = 3 to 8 embryo’s per genotype (WT N=3; K109*/+ N=5; K109*/K109* N=8) from 2 different litters. Two-way ANOVA followed by Tukey’s post-hoc test; ****p<0,0001. **B)** Bar plot showing the quantified ratios Aβ42/38 and Aβ42/40 from the Aβ profile in 3A from WT (black bars) and K109*/+ (orange bars) neurons. Two-tailed unpaired Student’s t-test. **C)** Representative western blot of the different γ-secretase complex subunits and APP substrate processing from *Psen1*^K109*^;App^*hu/hu*^ primary neurons. The blot on the left was cut at the 62kDa and 14kDa molecular weight bands. The upper membrane part was stained with Nicastrin antibodies (9C3), the middle part was stained with Psen1 CTF antibodies (Psen1-Loop CTF MAB5232) also recognizing Psen1 FL, and finally re-probed with antibodies against Actin-β (A5441). The lower membrane part was stained with antibodies against Pen2 (B126.2). The blot on the right was sequentially stained with Psen1-NTF (B19.3) which also recognizes Psen1-FL and with antibodies against APPC20 (B63.3) recognizing App FL and its carboxyterminal CTF stubs. Thereafter, the blot was cut at the 38kDa molecular weight marker and the upper part was stained with antibodies against Actin-β and the lower part was stained with antibodies against Psen2-CTF (D30G3). NuPAGE Bis-Tris 10% gels were used and all lanes were loaded with 20μg of protein. (WT N=3; K109*/+ N=5; K109*/K109* N=8). **D)** Bar plots showing the quantification of the different γ-secretase components and substrates from the western blot of *Psen1*^K109*^;App^*hu/hu*^ primary neurons in Figure 5C of WT (grey bars), K109* (orange bars) and K109*/K109* (red bars) neurons. One-way ANOVA followed by Tukey’s post-hoc test; *p<0,05; **p<0,01; ****p<0,0001. All data presented as Mean ± SEM.

We generated primary neuronal cultures from E15 embryonic littermates and measured endogenous soluble Aβ levels from conditioned media of the primary neuronal cultures using Elisa (Fig. 3A). Heterozygous *Psen1*^K109*/+^*;App*^*hu/hu*^ neurons compared to wild-type neurons showed no significant differences in Aβ production (Aβ38 :127 vs. 142 pg/ml; p = 0.57, Aβ40: 298 vs. 337 pg/ml; p = 0.62, Aβ42: 40 vs. 47 pg/ml; p = 0.57). There were no alterations in Aβ42/38 or Aβ42/40 ratios compared to wild-type neurons (0.30 vs 0.33 Aβ42/38 ratio; 0.13 vs. 0.14 Aβ42/40 ratio) (Fig. 3B). In homozygous *Psen1*^K109*/K109*^;App^*hu/hu*^ neurons Aβ38 and Aβ42 were below detection limits. Aβ40 was detectable but significantly reduced vs. wild-type neurons (30 vs. 337 pg/ml; p = 0.0004). We observed a 1.7-fold (p < 0.0001) increase in full length App (App-FL) and a 6.9-fold (p < 0.0001) increase in α- and β-secretase generated carboxyl fragments (CTF) of App in homozygous neurons (Fig. 3D).

We investigated *Psen1*^K109*^ expression by western blot of cell extracts from heterozygous and homozygous *Psen1*^K109*^ primary neurons (Fig. 3C and quantified in Fig. 3D). Heterozygous neurons showed a 50% reduction in Psen1-CTF levels, whereas in homozygous neurons no signal was detected. Intriguingly, we detected the expression of a genotype-dependent 12kDa truncated Psen1-NTF fragment (Psen1-NTF*) in hetero- and homozygous neurons only after long exposure (Supplementary Fig. 1A), indicating translation of the mRNA generated from the *Psen1*^K109*^ allele.

We investigated the impact of *Psen1*^K109*^ on the expression of its homologous gene Psen2, as well as on other -secretase subunits (Nicastrin, Pen2 and Aph1a or Aph1b) which together form the active proteolytic -secretase complexes(27). Our analysis revealed a slight but non-significant increase in Psen2 expression in heterozygous neurons, whereas homozygous *Psen1*^K109*^ neurons exhibited a significant 1.7-fold (p = 0.0017) increase, consistent with previous studies showing elevated Psen2 levels in knock out models(28).

We also observed a reduction in Pen2 levels, with heterozygous *Psen1*^K109*/+^ neurons exhibiting a 30% decrease versus WT levels (p = 0.0071) and homozygous neurons showing a drastic 80% reduction (p < 0.0001)(29). Nicastrin levels remained unchanged in heterozygous neurons but were significantly reduced to 60% of WT levels (p = 0.0067) in *Psen1*^K109*/109*^ neurons (Fig. 3D).

Overall, the heterozygous model, which most closely mimics the patient’s condition, shows significant effects on -secretase complex assembly. However, it does not impact overall Aβ processing, likely due to compensation by the wild-type allele. Thus, while there is clear evidence of a loss-of-function phenotype, this alone does not explain the accumulation of amyloid plaques observed in the patient.

**Figure 4:**
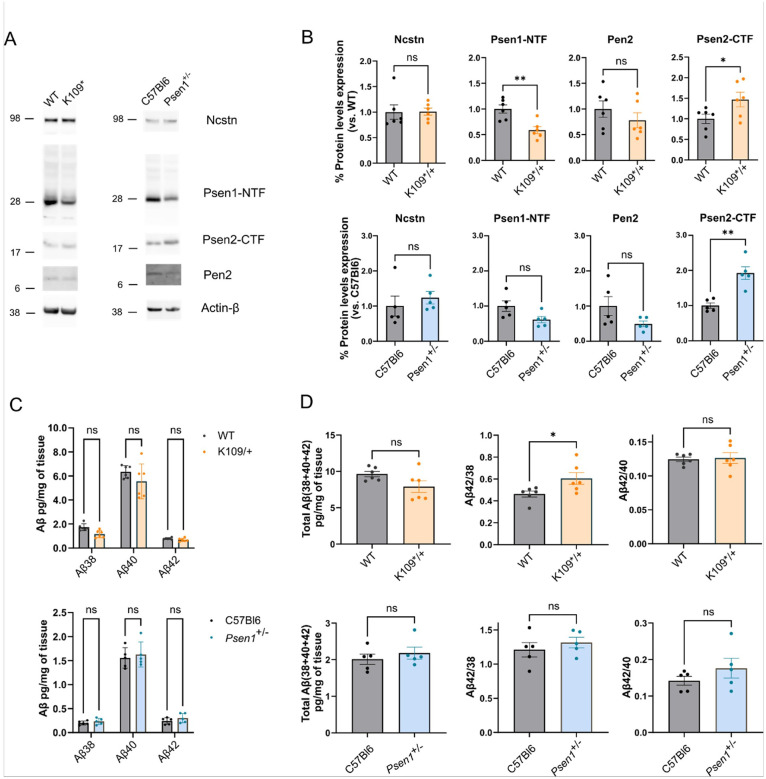
Comparison of *Psen1*^K109*/+^*;App*^*hu/hu*^ and *Psen1*^+/−^ aged heterozygous mice. **A)** Representative western blot from brain homogenate of ~20 months *Psen1*^K109*/+^*;App*^*hu/hu*^ (K109*/+) and *Psen1*^+/−^*;App*^*WT*^ versus their control mice *Psen1*^*WT*^*;App*^*hu/hu*^ (WT) and *Psen1*^*WT*^*; App*^*WT*^ (C57Bl6) respectively, showing the expression of different γ-secretase complex subunits. The membrane was cut at the 62kDa and 14kDa molecular weight bands. The upper membrane part was stained with antibodies against Nicastrin (9C3), the middle part was stained with antibodies against Psen1 NTF (B19.3) and then reprobed with antibodies against Psen2-CTF (D30G3). The lower part was stained with antibodies against Pen2 (B126.2). NuPAGE Bis-Tris 4–12% gels were used and all lanes were loaded with 30μg of protein. Actin-β (A5441) staining was done as loading control. Blots representative for N=5–6 mice. All samples were loaded in one blot. **B)** Bar plots showing the quantification of the protein levels of different γ-secretase components from the western blot in 4A. *Psen1*^K109*/+^*;App*^*hu/hu*^ (orange bars) and *Psen1*^+/−^*;App*^*WT*^ (blue bars) versus their control mice *Psen1*^*WT*^*;App*^*hu/hu*^ and *Psen1*^*WT*^*;App*^*WT*^ (C57Bl6 - grey bars) respectively. N=5–6 mice; Unpaired T-test; *p<0,05; **p<0,01. **C)** Bar plots showing the Aβ profile measured by MSD ELISA from brain homogenate of *Psen1*^K109*/+^*;App*^*hu/hu*^ (orange bars) and *Psen1*^+/−^*;App*^*WT*^ (blue bars) versus their control mice *Psen1*^*WT*^*;App*^*hu/hu*^ and *Psen1*^*WT*^*;App*^*WT*^ (grey bars) respectively. N=5–6 mice; 2way ANOVA followed by Šidák’s multiple comparison test; *p<0,05. **D)** Bar plots showing total Aβ (38+40+42) and ratios Aβ42/38 and Aβ42/40 from brain homogenate tissue of 18–20 months *Psen1*^K109*/+^*;App*^*hu/hu*^ (orange bars) and *Psen1*^+/−^*;App*^*WT*^ (blue bars) versus their control mice *Psen1*^*WT*^*;App*^*hu/hu*^ and *Psen1*^*WT*^*;App*^*WT*^ (grey bars) respectively N=5–6 mice; Unpaired T-test; *p<0,05. All data presented as Mean ± SEM.

We aged heterozygous *Psen1*^K109*/+^*;App*^*hu/hu*^ mice to ~ 20 months to investigate potential age x genotype interactions (Fig. 4A). In brain homogenates, we detected a 0.6-fold (p = 0.0028) reduction in Psen1 levels accompanied by a 1.5-fold increase (p = 0.0493) in Psen2 (Fig. 4B). However, Nicastrin and Pen2 levels remained unchanged (Fig. 4B). We were unable to detect the 12kDa Psen1-NTF truncated fragment found in the primary neurons, in brain extracts from both 6.5 months-old (Supplementary Fig. 1B) and 18 months-old animals (Supplementary Fig. 1C). We also investigated how the *Psen1*^K109*^ variant affects Aβ profiles with ageing. We observed a non-significant reduction in Aβ38 levels (0.6 vs. 0.9pg/mg; p = 0.41), Aβ40 (2.8 vs. 3.2 pg/mg; p = 0.13) and Aβ42 (0.3 vs. 0.4pg/mg; p = 0.99) peptides in *Psen1*^K109*/+^*;App*^*hu/hu*^ (Fig. 4C). Similar to our findings in primary neurons, Aβ43 levels were below detection limits (data not shown). Total Aβ production and the Aβ42/40 ratio were unaffected whereas the Aβ42/38 ratio showed a slight increase (0.6 vs. 0.5; p = 0.04) (Fig. 4D, Supplementary Fig. 2A). Notably, no Aβ plaque deposition was detected in 20 months-old brains as shown by immunohistochemistry (Supplementary Fig. 2B). To further assess the impact of Psen1 haploidy, we compared Aβ levels between wild-type (C57Bl6) and *Psen1*^+/−^ mice. No significant differences were observed, confirming that in loss-of-function mutations, Aβ production is completely compensated by the wild type allele(7) or by PSEN2 expression (Figs. 4C–4D).

### Human PSEN1 p.K109* variant generates aberrant N- and C-terminal fragments under overexpression conditions.

**Figure 5:**
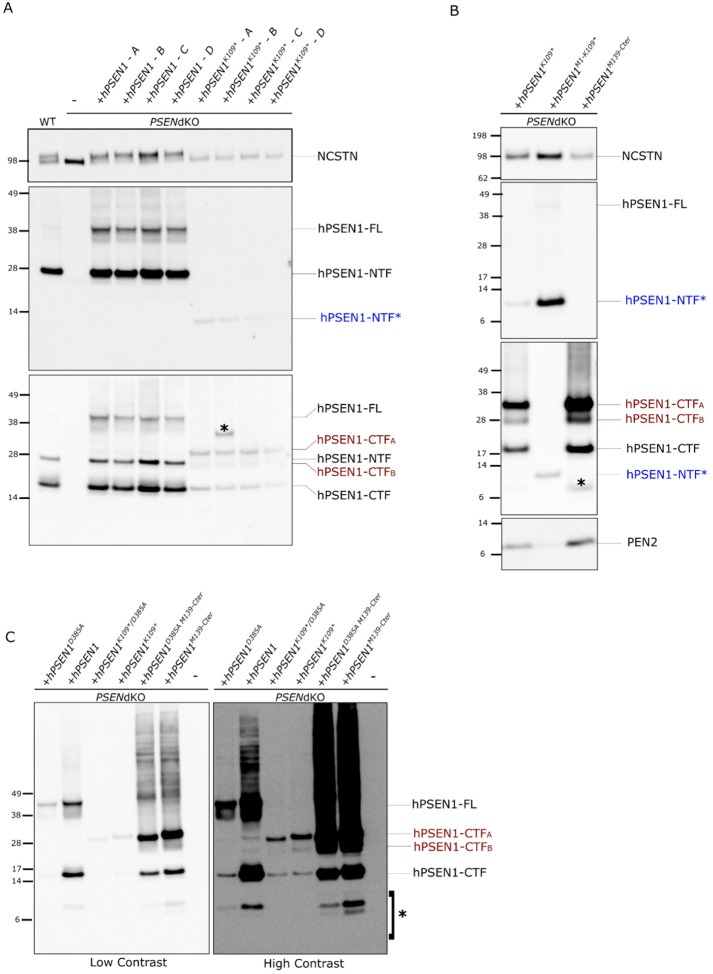
*hPSEN1*^K109^* short fragments do not reconstitute γ-secretase enzymatic activity. **A)** Western blot showing *PSENdKO* HeLa cells stably transduced with *hPSEN1* or *hPSEN1*^K109*^. Four pools of stably transduced HeLa *PSENdKO* lines were generated for rescue with *hPSEN1* WT or *hPSEN1*^K109*^ (pool A to D) for each gene as indicated. The upper panel was stained with an antibody against NCSTN (9C3). In the first lane an extract from WT HeLa cells is applied to demonstrate the two bands of NCSTN in normal culture conditions. In the second lane it is shown that NCSTN is not matured and runs as a single band in *PSENdKO*. Maturation is restored in the clones expressing the *hPSEN1*, but not in the cells transduced with *hPSEN1*^K109*^. The middle panel was stained with an antibody against hPSEN1-NTF (MKAD3.5) also recognizing hPSEN1-FL. Under overexpression conditions more full length PSEN1 is seen than in the control lane. The N-terminal fragment of ~12kDa generated with *hPSEN1*^K109*^ is indicated in blue (hPSEN1-NTF*). The lower panel shows the same blot (middle panel) re-stained with a hPSEN1-CTF (PSEN1-Loop CTF, MAB5232) antibody also recognizing hPSEN1 FL. On top of the N-terminal fragments seen in the middle panel, notice the normal PSEN1-CTF band that runs at ~28kDa in the first (control) lane and the cells transfected with *hPSEN1*. In lanes showing cells that express *hPSEN1*^K109*^ additional CTF bands are observed. We suppose that the hPSEN1-CTF_A_ (~30 kDa) is generated from ATG139, while the hPSEN1-CTF_B_ (~27 kDa) might be generated from the ATG146. The aberrant A and B hPSEN1 CTFs are indicated in red. Unexplained bands are indicated with an asterisk (*). NuPAGE Bis-Tris 4–12% gels were used and all lanes were loaded with 20 μg of protein. Ponceau S staining was done as loading control. **B)** Western blot showing *PSENdKO* Hela cells stably transfected with *hPSEN1*, *hPSEN1*^K109*^, *hPSEN1*^M1-K109*^ and *hPSEN1*^*M139-Cter*^. The upper panel was stained with an antibody against NCSTN (9C3). The second panel was stained with hPSEN1-NTF (MKAD3.5) antibody also recognizing hPSEN1 FL and the shorter ~12kDa PSEN1-NTF fragment indicated with a blue arrowhead. The third panel is the same as the middle panel but re-stained with a hPSEN1-CTF (D39D1) antibody recognizing hPSEN1 FL and the short A and B PSEN1-CTF fragments indicated in red. In blue is indicated the remaining signal from the previous staining corresponding to the PSEN1-NTF* ~12kDa band. Unknown bands are indicated with an asterisk (*). Samples were loaded on a second gel and stained with an antibody against PEN2 (B126.2). NuPAGE Bis-Tris 4–12% gels were used and all lanes were loaded with 25μg of protein. Ponceau S staining was done as loading control. **C)** Representative western blot of *PSENdKO* Hela cell lines stably transfected with *hPSEN1*, *hPSEN1*^K109*^ or *hPSEN1*^*M139-Cter*^ alone or in combination with the D385A mutation. The membrane was stained in the reverse order as in 5A and 5B. First with an antibody against PSEN1 CTF (D39D1) allowing the detection of hPSEN1 FL, hPSEN1 CTF and the shorter hPSEN1 CTF_A_ and CTF_B_ fragments, indicated in red. Membrane was re-probed with an antibody against PSEN1 NTF (MKAD3.5) (see Supplementary Figure 4B). Unknown bands are indicated with an asterisk (*). NuPAGE Bis-Tris 4–12% gels were used and all lanes were loaded with 25μg of protein. Ponceau S staining was done as loading control.

Our findings in mice demonstrated the generation of an aberrant ~ 12 kDa Psen1 fragment associated with the presence of the mutated allele (Supplementary Fig. 1). This fragment likely corresponds to a truncated protein translated from the initial start codon up to the premature stop codon TAG at position 109. To determine whether a similar protein fragment is produced from the human sequence, we transduced *PSEN1*&2^−/−^ double knock out (*PSENdKO*) HeLa cells to express either wild-type human *PSEN1* (*hPSEN1*) or mutant *hPSEN1*^K109*^(*PSEN1* c.325A > T). *PSENdKO* cells lack mature -secretase complexes. As expected, reintroducing wild type *PSEN1* restored γ-secretase complex formation, confirmed by the double bands indicating maturation of NCSTN(17) (Fig. 5A, upper panel). Immunostaining with N-terminal and C-terminal loop antibodies confirmed proper expression, endoproteolytic maturation, and the formation of stable wild type hPSEN1-NTF and -CTF (Fig. 5A, middle and lower panels). In contrast, expressing *hPSEN1*^K109*^ failed to support maturation of the γ-secretase complex (Fig. 5A, upper panel). However, staining with the hPSEN1 N-terminal antibody revealed the weak expression of a ~ 12 kDa band (hPSEN1-NTF*; Fig. 5A upper panel), which may correspond to the protein fragment seen in the mouse studies.

Unexpectedly, staining the same blot with a PSEN1 C-terminal specific antibody (Fig. 5A lower panel) demonstrated that the truncated mutant generated PSEN1-CTF fragments with an estimated mobility of ~ 17kDa, similar to the control, besides additional C-terminal fragments, of ~ 30kDa (PSEN1-CTF_A_) and ~ 27kDa (PSEN1-CTF_B_) (red; Fig. 5A).

Further inspection of the PSEN1 sequence revealed two internal ATG codons, located at positions 139 and 146 (Supplementary Fig. 3). We constructed two cDNAs, M1-K109* (~ 12kDa) and M139-Cter (~ 37kDa), with the former starting at the first ATG and ending at the stop codon in position K109 introduced by the c.325A > T mutation, and the latter starting at the second internal ATG and ending at the canonical stop codon, respectively. We transfected these cDNAs into *PSENdKO* HeLa cells and compared the protein fragments generated in these cells with those observed in the cells transfected with full *hPSEN1*^K109*^ (Fig. 5B). *PSENdKO* HeLa cells overexpressing *hPSEN1*^M1 − K109*^ produced a protein matching the mobility of the ~ 12kDa N-terminal fragment (blue; Fig. 5B) observed in *hPSEN1*^K109*^ transfected cells. *PSENdKO* HeLa cells overexpressing the *hPSEN1*^*M139 − Cter*^ fragment produced a ~ 17kDa C-terminal fragment, also present in the *hPSEN*^K109*^ condition, along with the two additional PSEN1 C-terminal fragments A and B (indicated in red; Fig. 5B). These results suggest that PSEN1–CTF_A_ is generated from the ATG at codon position 139, while PSEN1-CTF_B_ is likely a proteolytic fragment of the M146-Cter derived protein. As is shown in Fig. 5B, neither full length *hPSEN1*^K109*^ nor the two truncated constructs supported γ-secretase complex maturation as indicated by the absence of NCSTN glycosylation. Additionally, PEN2 expression was decreased in *hPSEN1*^K109*^ transfected cells, nearly abolished in *hPSEN1*^M1 − K109*^ cells and preserved in *hPSEN1*^*M139 − Cter*^. This is consistent with previous studies demonstrating that PSEN1 is required for PEN2 stabilization(30, 31) and it suggests that the C-terminal fragment generated from the second ATG may interact with PEN2.

### Human PSEN1 p.K109* variant and its fragments do not confer γ-Secretase activity

To evaluate whether these PSEN1-derived fragments could restore γ-secretase enzymatic activity, we transiently overexpressed a synthetic APP_C99_ substrate fused to GFP at its C-terminus allowing visualization of cleavage products. Neither *hPSEN1*^K109*^ nor its truncated fragments (*hPSEN1*^M1 − K109*^ or *hPSEN1*^*M139 − Cter*^) confer measurable activity to the complex as evidenced by the absence of Aβ peptides (Aβ38, Aβ40, Aβ42, and Aβ43) in the conditioned media of transfected cells as measured by MSD Elisa (Supplementary Fig. 4A) and the lack of AICD production (data not shown). Thus, under these overexpression conditions, the *hPSEN1*^K109*^ variant and its fragments result in a complete loss of Aβ generation.

Intriguingly, *hPSEN1*^*M139 − Cter*^ retains the two catalytic aspartate residues (see Supplementary Fig. 3) and restores PEN2 expression (Fig. 5A). We also observed a ~ 17kDa protein band with mobility similar to the normal PSEN1-CTF, raising the question whether this C-terminal fragment could be generated via auto-catalysis. To test this, we introduced a mutation replacing Asp^385^ with alanine (D385A) in the *hPSEN1*^*M139 − Cter*^ (Fig. 5C). The D385A mutation renders -secretase catalytically inactive, thereby preventing PSEN1 endoproteolysis in wild type conditions(9, 32). We then transiently transfected *PSENdKO* HeLa cells with wild-type *hPSEN1*, *hPSEN1*^K109*^, *hPSEN1*^*M139 − Cter*^ and hPSEN1^D385A M139−Cter^ (Fig. 5C). As expected, *hPSEN1* expression led to the formation of full length PSEN1 (FL), and its proteolytic NTF (Supplementary Fig. 4B) and CTF fragments (Fig. 5C). *hPSEN1*^*D385A*^ alone resulted in PSEN1-FL expression due to suppression of endoproteolysis. However, it also produced a fragment running at the same level as the NTF (Supplementary Fig. 4B) and CTF fragments of presenilin, which we have previously observed in overexpressed *hPSEN1*^*D385A*^ (30). Importantly, introducing the D385A mutation into *hPSEN1*^K109*^ or *hPSEN1*^*M139 − Cter*^ did not alter the levels of NTF (Supplementary Fig. 4B) or CTF fragments (Fig. 5C). Based on our previous findings, this suggests that alternative proteases can cleave these same sites when -secretase is inactive(30, 33, 34).

## Discussion

Here we describe the first *PSEN1* nonsense variant associated with the occurrence of AD. Both the index case and his father carried a heterozygous *PSEN1* c.325A > T variant, leading to the substitution of lysine 109 with a premature stop-gain codon (p.K109*) resulting in a severe truncation of the protein. Amyloid plaques were detected in both subjects and the overall clinical work-up indicates that these two patients suffered from AD. Despite in depth investigation of this mutation in a mouse model and in human transfected cells it remains unclear how this mutation affects amyloid peptide generation and could be linked to amyloid plaque formation. While the clinical observations, demonstrating EOAD in both index patient and father, suggest that the disease is associated with this Presenilin loss of function mutation (LOF) mutation, the case is illustrative of the challenges clinicians and geneticists are confronted with when having to determine whether a novel *PSEN1* mutation can be causally linked to the disease.

To date, only three other heterozygous *PSEN1* stop-gain mutations, resulting in a truncated PSEN1 protein, have been documented, but neither of them has been proposed to cause AD. PSEN1 p.W294* was associated with acute encephalopathy and retinitis pigmentosa(35), while *PSEN1* p.P242LfsX11 was associated with the dermatological condition *hidradenitis suppurativa*(36). It is of note that the index patient described herein had an autoimmune disease affecting joints and skin (psoriatic arthritis) but this is quite distinct from *hidradenitis suppurativa* and its relevance is unclear. *PSEN1* p.S357*, was reported in a single patient with cerebral amyloid angiopathy and cognitive decline. However, this patient also carried a known pathogenic *PSEN1* p.R377W variant(37), making it difficult to draw conclusions regarding the disease-causing effect of the *PSEN1* p.S357* variant alone. In fact, more than 190 mutations occurring in *PSEN1* that cause AD are missense mutations, encoding a presenilin protein that will decrease processivity shifting the Aβ profile to generate long Aβ (> 40 aa)(2, 12, 38).

Interestingly, the *PSEN2* p.K115Efs*11 variant, found in two unrelated families diagnosed with EOAD(39, 40), occurs at the same position as the variant p.K109*. The *PSEN2* K115Efs*11 mutation induces aberrant alternative splicing events that may partially restore the reading frame and produce altered protein isoforms(40, 41). However, *PSEN1* undergoes less alternative splicing(42) and unlike *PSEN2*, which may tolerate or even facilitate alternative splicing under pathological conditions, *PSEN1* appears less amenable to such compensatory mechanisms(42).

Large-scale exome studies reveal that *PSEN1* is highly intolerant to LOF mutations. In the gnomAD v4.1.0 dataset of 730,947 exomes, only 13 rare qualifying LOF single nucleotide variants (SNVs) are observed in the canonical transcript, all as heterozygotes(43), far fewer than expected, indicating strong selective constraint. This is quantified by a pLI (probability of loss-of-function intolerance) score, which ranges from values near 1 indicating intolerance to LOF variants to values near 0 reflecting complete tolerance. *PSEN1* has a pLI score ranging from 0.97 to 1.00 in gnomAD, even when excluding neuropsychiatric study participants, confirming its high intolerance to LOF mutations. For comparison, *APP* also shows a pLI score of 1, while *PSEN2*, with a score of 0, is tolerant to LOF mutations. The scarcity of LOF variants in *PSEN1* and *APP* suggests they negatively impact reproductive fitness, limiting their transmission. Moreover, *PSEN1’s* high pLI score, similar to genes involved in pediatric neurological disorders (e.g., *PTEN*), implies potential effects on critical biological pathways influencing both neurological development and reproductive fitness, highlighting its evolutionary significance.

To establish causality, we generated a knock-in mouse model mimicking the PSEN1 c.325A > T variant and crossed it with *App*^*hu/hu*^ mouse(18) to assess Aβ alterations. Homozygous mice exhibited severe -secretase loss of function, resembling homozygous *Psen1 KO* mice(7, 8, 25), with impaired Notch signaling, reduced Aβ production and APP-CTF accumulation, confirming the mutation’s deleterious impact(7). This was further supported by disrupted -secretase assembly, evidenced by absent PSEN1 heterodimers, low PEN2 expression, and defective Nicastrin maturation, as seen in previously characterized *Psen1* knock out models(7, 25, 44).

Heterozygous mice, more reflective of patient conditions, showed no major Notch defects but displayed mild biochemical abnormalities, including reduced Pen2 expression and slight App-CTF accumulation. Surprisingly, no significant changes to Aβ or amyloid plaques were detected, even in aged mice, except for a slight reduction in Aβ38 levels. Unlike *PSEN1* mutations linked to EOAD of autosomal dominant inheritance, this mutation did not increase the short/long Aβ ratio (12, 13, 45, 46).

Notably, aged mice exhibited increased Psen2 expression, suggesting a compensatory mechanism for Psen1 loss(31). Given that PSEN2 complexes generate less Aβ38 than PSEN1(47, 48), this shift may create a more aggregation-prone environment(49), potentially contributing to disease pathology. Disruptions in PSEN2 function could impair endolysosomal proteostasis and exacerbate neurodegenerative processes(50), warranting further investigation.

In human overexpression cell models, the *PSEN1*^K109*^ mutation produced two unexpected protein fragments, one of which retained the γ-secretase catalytic domain but lacked enzymatic activity. While these fragments may interact with the γ-secretase complex, they do not restore function.

Our findings indicate that the *PSEN1* c.325A > T (p.K109*) variant is not a straightforward loss-of-function mutation, as it generates two protein fragments with unknown effects. However, despite extensive *in vivo* and *in vitro* characterization, we cannot definitively classify it as a causal pathogenic variant. This case highlights the complexity of genetic risk assessment in AD and does not refute the prevailing consensus that the hundreds of previously identified *PSEN1* missense, insertion, and deletion mutations are directly linked to EOAD through well-established effects on Aβ processing(12, 38, 45, 51, 52).

## Conclusions

More broadly, this study underscores the need for a comprehensive approach to genetic analysis in AD. While known pathogenic variants remain key diagnostic markers, a broader exploration of the genetic landscape—including potential compensatory mechanisms and variant interactions—could provide deeper insights into disease mechanisms(45, 53). Integrating multiple genetic factors will be essential for refining diagnostic strategies and advancing precision medicine in neurodegenerative disorders.

## Supplementary Material

This is a list of supplementary files associated with this preprint. Click to download.

- PS1K109SupplementaryFiguresTables.docx
- PS1K109SupplementaryfileMolNeurodegAdv.docx

## Figures and Tables

**Table 1:** Amyloid beta and Tau profile in CSF of the index patient, carrier of the *PSEN1*^K109*^ variant.

CSF	Index patient	Non-mutation carrying controls (N=31)	Mutation-carrying controls (n = 27)
Aβ42 (S.D.)	454 pg/ml	890 (329) pg/ml	356 (114) pg/ml
Aβ40 (S.D.)	6911 pg/ml	9434 (3897) pg/ml	7848 (2475) pg/ml
Ratio Aβ42/40	0.066	0.094	0.045
Total tau (S.D.)	123 pg/ml *	52 (19) pg/ml*	158 (70) pg/ml
P-Tau (S.D.)	115 pg/ml *	31 (11) pg/ml*	91 (34) pg/ml

* Significant difference by two-tailed unpaired Students’s t-test, p ≤ 0.001

**Table 2:** Amyloid beta profile in plasma of the index patient, carrier of the the *PSEN1*^K109*^ variant.

Plasma	Index patient	Non-mutation carrying controls (N=40)	Mutation-carrying controls (n = 31)
Aβ42 (S.D.)	47 pg/ml	36 (7.3) pg/ml	45 (14) pg/ml
Aβ40 (S.D.)	138 pg/ml	152 (31) pg/ml	147 (42) pg/ml
Ratio Aβ42/40	0.34	0.24	0.30

There were no significant differences by two-tailed unpaired Students’s t-test.

## Data Availability

All data and materials generated or analyzed during this study are available from the corresponding author on reasonable request.

## Abbreviations

Aβ: amyloid-β
ADAD: autosomal dominant Alzheimer’s disease
AD: Alzheimer’s disease
APP: amyloid precursor protein
CDR: clinical dementia rating
CSF: cerebrospinal fluid
CTF: C-terminal fragment
EOAD: early-onset Alzheimer’s disease
FL: full length
LOF: loss of function
MRI: magnetic resonance imaging
NTF: N-terminal fragment
PET: positron emission tomography
PSEN: presenilin
SEM: standard error mean
TM: transmembrane domain
WT: wild-type

## References

[1] Campion D, Dumanchin C, Hannequin D, Dubois B, Belliard S, Puel M, Early-onset autosomal dominant Alzheimer disease: prevalence, genetic heterogeneity, and mutation spectrum. Am J Hum Genet. 1999;65(3):664–70. 10441572 10.1086/302553 PMC1377972

[2] De Strooper B, Karran E. New precision medicine avenues to the prevention of Alzheimer’s disease from insights into the structure and function of gamma-secretases. EMBO J. 2024;43(6):887–903. 38396302 10.1038/s44318-024-00057-w PMC10943082

[3] Ringman JM, Goate A, Masters CL, Cairns NJ, Danek A, Graff-Radford N, Genetic heterogeneity in Alzheimer disease and implications for treatment strategies. Curr Neurol Neurosci Rep. 2014;14(11):499. 25217249 10.1007/s11910-014-0499-8 PMC4162987

[4] Ryan NS, Nicholas JM, Weston PSJ, Liang Y, Lashley T, Guerreiro R, Clinical phenotype and genetic associations in autosomal dominant familial Alzheimer’s disease: a case series. Lancet Neurol. 2016;15(13):1326–35. 27777022 10.1016/S1474-4422(16)30193-4

[5] Bergmans BA, De Strooper B. gamma-secretases: from cell biology to therapeutic strategies. Lancet Neurol. 2010;9(2):215–26. 20129170 10.1016/S1474-4422(09)70332-1

[6] Bateman RJ, Aisen PS, De Strooper B, Fox NC, Lemere CA, Ringman JM, Autosomal-dominant Alzheimer’s disease: a review and proposal for the prevention of Alzheimer’s disease. Alzheimers Res Ther. 2011;3(1):1. 21211070 10.1186/alzrt59 PMC3109410

[7] De Strooper B, Saftig P, Craessaerts K, Vanderstichele H, Guhde G, Annaert W, Deficiency of presenilin-1 inhibits the normal cleavage of amyloid precursor protein. Nature. 1998;391(6665):387–90. 9450754 10.1038/34910

[8] De Strooper B, Annaert W, Cupers P, Saftig P, Craessaerts K, Mumm JS, A presenilin-1-dependent gamma-secretase-like protease mediates release of Notch intracellular domain. Nature. 1999;398(6727):518–22. 10206645 10.1038/19083

[9] Wolfe MS, Xia W, Ostaszewski BL, Diehl TS, Kimberly WT, Selkoe DJ. Two transmembrane aspartates in presenilin-1 required for presenilin endoproteolysis and gamma-secretase activity. Nature. 1999;398(6727):513–7. 10206644 10.1038/19077

[10] Struhl G, Greenwald I. Presenilin is required for activity and nuclear access of Notch in Drosophila. Nature. 1999;398(6727):522–5. 10206646 10.1038/19091

[11] Li YM, Lai MT, Xu M, Huang Q, DiMuzio-Mower J, Sardana MK, Presenilin 1 is linked with gamma-secretase activity in the detergent solubilized state. Proc Natl Acad Sci U S A. 2000;97(11):6138–43. 10801983 10.1073/pnas.110126897 PMC18571

[12] Szaruga M, Munteanu B, Lismont S, Veugelen S, Horré K, Mercken M, Alzheimer’s-Causing Mutations Shift Aβ Length by Destabilizing γ-Secretase-Aβn Interactions. Cell. 2017;170(3):443–56.e14. 28753424 10.1016/j.cell.2017.07.004

[13] Sherrington R, Rogaev EI, Liang Y, Rogaeva EA, Levesque G, Ikeda M, Cloning of a gene bearing missense mutations in early-onset familial Alzheimer’s disease. Nature. 1995;375(6534):754–60. 7596406 10.1038/375754a0

[14] Szaruga M, Veugelen S, Benurwar M, Lismont S, Sepulveda-Falla D, Lleo A, Qualitative changes in human γ-secretase underlie familial Alzheimer’s disease. J Exp Med. 2015;212(12):2003–13. 26481686 10.1084/jem.20150892 PMC4647268

[15] Kosik KS, Munoz C, Lopez L, Arcila ML, Garcia G, Madrigal L, Homozygosity of the autosomal dominant Alzheimer disease presenilin 1 E280A mutation. Neurology. 2015;84(2):206–8. 25471389 10.1212/WNL.0000000000001130 PMC4336083

[16] Parker J, Mozaffar T, Messmore A, Deignan JL, Kimonis VE, Ringman JM. Homozygosity for the A431E mutation in PSEN1 presenting with a relatively aggressive phenotype. Neurosci Lett. 2019;699:195–8. 30716424 10.1016/j.neulet.2019.01.047 PMC7759143

[17] Herreman A, Van Gassen G, Bentahir M, Nyabi O, Craessaerts K, Mueller U, gamma-Secretase activity requires the presenilin-dependent trafficking of nicastrin through the Golgi apparatus but not its complex glycosylation. J Cell Sci. 2003;116(Pt 6):1127–36. 12584255 10.1242/jcs.00292

[18] Serneels L, T’Syen D, Perez-Benito L, Theys T, Holt MG, De Strooper B. Modeling the β-secretase cleavage site and humanizing amyloid-beta precursor protein in rat and mouse to study Alzheimer’s disease. Mol Neurodegener. 2020;15(1):60. 33076948 10.1186/s13024-020-00399-z PMC7574558

[19] Livak KJ, Schmittgen TD. Analysis of relative gene expression data using real-time quantitative PCR and the 2(-Delta Delta C(T)) Method. Methods. 2001;25(4):402–8. 11846609 10.1006/meth.2001.1262

[20] Esselens C, Oorschot V, Baert V, Raemaekers T, Spittaels K, Serneels L, Presenilin 1 mediates the turnover of telencephalin in hippocampal neurons via an autophagic degradative pathway. J Cell Biol. 2004;166(7):1041–54. 15452145 10.1083/jcb.200406060 PMC2172014

[21] Annaert WG, Esselens C, Baert V, Boeve C, Snellings G, Cupers P, Interaction with telencephalin and the amyloid precursor protein predicts a ring structure for presenilins. Neuron. 2001;32(4):579–89. 11719200 10.1016/s0896-6273(01)00512-8

[22] Serneels L, Bammens L, Zwijsen A, Tolia A, Chavez-Gutierrez L, De Strooper B. Functional and topological analysis of PSENEN, the fourth subunit of the gamma-secretase complex. J Biol Chem. 2024;300(1):105533. 38072061 10.1016/j.jbc.2023.105533 PMC10790097

[23] Morris JC. Clinical dementia rating: a reliable and valid diagnostic and staging measure for dementia of the Alzheimer type. Int Psychogeriatr. 1997;9 Suppl 1:173–6; discussion 7–8. 9447441 10.1017/s1041610297004870

[24] Donoviel DB, Hadjantonakis AK, Ikeda M, Zheng H, Hyslop PS, Bernstein A. Mice lacking both presenilin genes exhibit early embryonic patterning defects. Genes Dev. 1999;13(21):2801–10. 10557208 10.1101/gad.13.21.2801 PMC317124

[25] Shen J, Bronson RT, Chen DF, Xia W, Selkoe DJ, Tonegawa S. Skeletal and CNS defects in Presenilin-1-deficient mice. Cell. 1997;89(4):629–39. 9160754 10.1016/s0092-8674(00)80244-5

[26] Wong PC, Zheng H, Chen H, Becher MW, Sirinathsinghji DJ, Trumbauer ME, Presenilin 1 is required for Notch1 and DII1 expression in the paraxial mesoderm. Nature. 1997;387(6630):288–92. 9153393 10.1038/387288a0

[27] De Strooper B. Aph-1, Pen-2, and Nicastrin with Presenilin generate an active gamma-Secretase complex. Neuron. 2003;38(1):9–12. 12691659 10.1016/s0896-6273(03)00205-8

[28] Stanga S, Vrancx C, Tasiaux B, Marinangeli C, Karlstrom H, Kienlen-Campard P. Specificity of presenilin-1- and presenilin-2-dependent gamma-secretases towards substrate processing. J Cell Mol Med. 2018;22(2):823–33. 28994238 10.1111/jcmm.13364 PMC5783875

[29] Steiner H, Winkler E, Edbauer D, Prokop S, Basset G, Yamasaki A, PEN-2 is an integral component of the gamma-secretase complex required for coordinated expression of presenilin and nicastrin. J Biol Chem. 2002;277(42):39062–5. 12198112 10.1074/jbc.C200469200

[30] Nyabi O, Bentahir M, Horre K, Herreman A, Gottardi-Littell N, Van Broeckhoven C, Presenilins mutated at Asp-257 or Asp-385 restore Pen-2 expression and Nicastrin glycosylation but remain catalytically inactive in the absence of wild type Presenilin. J Biol Chem. 2003;278(44):43430–6. 12885769 10.1074/jbc.M306957200

[31] Watanabe N, Tomita T, Sato C, Kitamura T, Morohashi Y, Iwatsubo T. Pen-2 is incorporated into the gamma-secretase complex through binding to transmembrane domain 4 of presenilin 1. J Biol Chem. 2005;280(51):41967–75. 16234244 10.1074/jbc.M509066200

[32] Kimberly WT, Xia W, Rahmati T, Wolfe MS, Selkoe DJ. The transmembrane aspartates in presenilin 1 and 2 are obligatory for gamma-secretase activity and amyloid beta-protein generation. J Biol Chem. 2000;275(5):3173–8. 10652302 10.1074/jbc.275.5.3173

[33] Loetscher H, Deuschle U, Brockhaus M, Reinhardt D, Nelboeck P, Mous J, Presenilins are processed by caspase-type proteases. J Biol Chem. 1997;272(33):20655–9. 9252383 10.1074/jbc.272.33.20655

[34] Hansson CA, Popescu BO, Laudon H, Cedazo-Minguez A, Popescu LM, Winblad B, Caspase cleaved presenilin-1 is part of active gamma-secretase complexes. J Neurochem. 2006;97(2):356–64. 16539675 10.1111/j.1471-4159.2006.03735.x

[35] You C, Zeng W, Deng L, Lei Z, Gao X, Zhang VW, Identification and Clinical Analysis of the First Nonsense Mutation in the PSEN1 Gene in a Family With Acute Encephalopathy and Retinitis Pigmentosa. Front Neurol. 2020;11:319. 32431660 10.3389/fneur.2020.00319 PMC7214681

[36] Wang B, Yang W, Wen W, Sun J, Su B, Liu B, Gamma-secretase gene mutations in familial acne inversa. Science. 2010;330(6007):1065. 20929727 10.1126/science.1196284

[37] Palmieri I, Valente M, Farina LM, Gana S, Minafra B, Zangaglia R, PSEN1 Compound Heterozygous Mutations Associated with Cerebral Amyloid Angiopathy and Cognitive Decline Phenotype. Int J Mol Sci. 2021;22(8). 10.3390/ijms22083870 PMC8069161 33918046

[38] Chávez-Gutiérrez L, Bammens L, Benilova I, Vandersteen A, Benurwar M, Borgers M, The mechanism of γ-Secretase dysfunction in familial Alzheimer disease. EMBO J. 2012;31(10):2261–74. 22505025 10.1038/emboj.2012.79 PMC3364747

[39] Jayadev S, Leverenz JB, Steinbart E, Stahl J, Klunk W, Yu CE, Alzheimer’s disease phenotypes and genotypes associated with mutations in presenilin 2. Brain. 2010;133(Pt 4):1143–54. 20375137 10.1093/brain/awq033 PMC2850581

[40] Braggin JE, Bucks SA, Course MM, Smith CL, Sopher B, Osnis L, Alternative splicing in a presenilin 2 variant associated with Alzheimer disease. Ann Clin Transl Neurol. 2019;6(4):762–77. 31020001 10.1002/acn3.755 PMC6469258

[41] Hin N, Newman M, Kaslin J, Douek AM, Lumsden A, Nik SHM, Accelerated brain aging towards transcriptional inversion in a zebrafish model of the K115fs mutation of human PSEN2. PLoS One. 2020;15(1):e0227258. 31978074 10.1371/journal.pone.0227258 PMC6980398

[42] Course MM, Gudsnuk K, Keene CD, Bird TD, Jayadev S, Valdmanis PN. Aberrant splicing of PSEN2, but not PSEN1, in individuals with sporadic Alzheimer’s disease. Brain. 2023;146(2):507–18. 35949106 10.1093/brain/awac294 PMC10169283

[43] Gudmundsson S, Singer-Berk M, Watts NA, Phu W, Goodrich JK, Solomonson M, Variant interpretation using population databases: Lessons from gnomAD. Hum Mutat. 2022;43(8):1012–30. 34859531 10.1002/humu.24309 PMC9160216

[44] Uh K, Monarch K, Reese ED, Rodriguez K, Yoon J, Spate LD, Impaired Skeletal Development by Disruption of Presenilin-1 in Pigs and Generation of Novel Pig Models for Alzheimer’s Disease. J Alzheimers Dis. 2024;101(2):445–61. 39177593 10.3233/JAD-231297 PMC11492100

[45] Petit D, Fernandez SG, Zoltowska KM, Enzlein T, Ryan NS, O’Connor A, Abeta profiles generated by Alzheimer’s disease causing PSEN1 variants determine the pathogenicity of the mutation and predict age at disease onset. Mol Psychiatry. 2022;27(6):2821–32. 35365805 10.1038/s41380-022-01518-6 PMC9156411

[46] Schultz SA, Liu L, Schultz AP, Fitzpatrick CD, Levin R, Bellier J-P, Functional variations in gamma-secretase activity are critical determinants of the clinical, biomarker, and cognitive progression of autosomal dominant Alzheimer’s disease. bioRxiv. 2023:2023.07.04.547688.

[47] Acx H, Chavez-Gutierrez L, Serneels L, Lismont S, Benurwar M, Elad N, Signature amyloid beta profiles are produced by different gamma-secretase complexes. J Biol Chem. 2014;289(7):4346–55. 24338474 10.1074/jbc.M113.530907 PMC3924297

[48] Schmidt FC, Fitz K, Feilen LP, Okochi M, Steiner H, Langosch D. Different transmembrane domains determine the specificity and efficiency of the cleavage activity of the gamma-secretase subunit presenilin. J Biol Chem. 2023;299(5):104626. 36944398 10.1016/j.jbc.2023.104626 PMC10164903

[49] Moore BD, Martin J, de Mena L, Sanchez J, Cruz PE, Ceballos-Diaz C, Short Abeta peptides attenuate Abeta42 toxicity in vivo. J Exp Med. 2018;215(1):283–301. 29208777 10.1084/jem.20170600 PMC5748850

[50] Perdok A, Van Acker ZP, Vrancx C, Sannerud R, Vorsters I, Verrengia A, Altered expression of Presenilin2 impacts endolysosomal homeostasis and synapse function in Alzheimer’s disease-relevant brain circuits. Nat Commun. 2024;15(1):10412. 39613768 10.1038/s41467-024-54777-y PMC11607342

[51] Veugelen S, Saito T, Saido TC, Chavez-Gutierrez L, De Strooper B. Familial Alzheimer’s Disease Mutations in Presenilin Generate Amyloidogenic Abeta Peptide Seeds. Neuron. 2016;90(2):410–6. 27100199 10.1016/j.neuron.2016.03.010

[52] Le Guennec K, Veugelen S, Quenez O, Szaruga M, Rousseau S, Nicolas G, Deletion of exons 9 and 10 of the Presenilin 1 gene in a patient with Early-onset Alzheimer Disease generates longer amyloid seeds. Neurobiol Dis. 2017;104:97–103. 28461250 10.1016/j.nbd.2017.04.020

[53] Schultz SA, Liu L, Schultz AP, Fitzpatrick CD, Levin R, Bellier JP, gamma-Secretase activity, clinical features, and biomarkers of autosomal dominant Alzheimer’s disease: cross-sectional and longitudinal analysis of the Dominantly Inherited Alzheimer Network observational study (DIAN-OBS). Lancet Neurol. 2024;23(9):913–24. 39074479 10.1016/S1474-4422(24)00236-9 PMC11822357

